# Linking rivers, mixing faunas: How artificial connectivity between the Middle and Upper Paraná River basins shapes fish diversity in a tributary of the Itaipu Reservoir, Brazil

**DOI:** 10.1111/jfb.70249

**Published:** 2025-11-11

**Authors:** Lucas E. P. Kampfert, João C. Maicrovicz, Daniel R. Blanco, Denise Lange, Carla S. Pavanelli, Heleno Brandão

**Affiliations:** ^1^ Programa de Pós‐Graduação em Recursos Naturais e Sustentabilidade Universidade Tecnológica Federal do Paraná (UTFPR), Campus Santa Helena Santa Helena Brazil; ^2^ Curso de Licenciatura em Ciências Biológicas Universidade Tecnológica Federal do Paraná (UTFPR), Campus Santa Helena Santa Helena Brazil; ^3^ Núcleo de Pesquisas em Limnologia, Ictiologia e Aquicultura (Nupélia) Universidade Estadual de Maringá Maringá Brazil; ^4^ Programa de Pós‐Graduação em Ecologia de Ambientes Aquáticos Continentais Universidade Estadual de Maringá Maringá Brazil

**Keywords:** biogeography, Iguaçu River, Middle Paraná River, non‐native species, Upper Paraná River

## Abstract

This work aimed to investigate the distribution and occurrence of fish species along the São Francisco Falso Braço Norte (SFFBN) River basin, a tributary of the Middle Paraná River basin now artificially connected to the Upper Paraná ecoregion, to evaluate how such connectivity may affect the biogeographic distribution and regional composition of freshwater fish species. Quarterly samplings were conducted between August 2022 and May 2023 at 12 sampling points distributed along the longitudinal axis of the basin. The fish were collected using sieves, casts, seines and gillnets. The results suggest that, although the SFFBN River basin is currently integrated into the Upper Paraná ecoregion and strongly influenced by the dynamics of the Itaipu Reservoir, its fish community still harbours species likely linked to the biogeographic history of the Middle Paraná River basin. These species, particularly in the middle and upper stretches of the SFFBN River basin, appear to have persisted without being directly affected by the damming. Therefore, monitoring the fish community in Itaipu's tributaries is essential for understanding the impacts of ongoing ichthyofaunal homogenization between the Lower and Upper Paraná ecoregions.

## 1 INTRODUCTION

The complex South American drainage network harbours more than 5750 known fish species, and it is estimated that between 3000 and 4000 species will yet to be described (Cassemiro et al., 2023; Reis et al., 2016). This group corresponds to the most species‐rich continental vertebrate fauna on the planet (Albert & Reis, 2011). This continental fish diversity is composed of several regionally defined faunas, each one with somewhat distinct but overlapping taxonomic compositions (Albert et al., 2020). Unlike most terrestrial taxa, the evolution of freshwater fish has been strongly influenced by natural connecting hydrogeographic events within and between river basins that have shaped contemporary biogeography and diversity patterns (Albert et al., 2011; Cassemiro et al., 2023).

The La Plata River basin is the third most fish species‐rich basin in South America, with approximately 900 species and a high level of endemism (48%) (Reis et al., 2016). This basin can be divided into seven ecoregions based on the composition and distribution of species and ecological and evolutionary patterns: Upper Paraná, Lower Paraná, Iguassu, Paraguay, Chaco, Upper Uruguay and Lower Uruguay (Abell et al., 2008). Among these, the Upper Paraná and Iguassu ecoregions exhibit particularly high levels of endemism: 30% and 69%, respectively [Word Wildlife Fund (WWF), The Nature Conservancy (TNC), 2019; Mezzaroba et al., 2021]. This endemism is closely linked to evolutionary processes associated with natural geographic barriers, such as the Sete Quedas Falls (in the Upper Paraná ecoregion) and the Iguassu Falls (in the Iguassu ecoregion), which historically restricted upstream dispersal of fish populations (e.g., Sivasundar et al., 2001; Piálek et al., 2012, 2019; Machado et al., 2018; Wendt et al., 2019). In contrast, the Lower Paraná ecoregion supports a lower proportion of endemic species, estimated at approximately 15% [Word Wildlife Fund (WWF), The Nature Conservancy (TNC), 2019].

The boundary between the Upper and Lower Paraná ecoregions proposed by Abell et al. (2008) reflects the artificial biogeographic changes caused by the Itaipu Hydroelectric Power Plant construction. The filling of the Itaipu Reservoir in 1982 submerged the Sete Quedas Falls, which historically marked the biogeographic boundary between two distinct hydrographic units: the Upper Paraná River basin, located upstream of Sete Quedas Falls (sensu Bonetto, 1986), and the Middle Paraná River basin, defined as the stretch of the Paraná River and its tributaries between Sete Quedas Falls and Apipé Falls (sensu Říčan et al., 2019). As a consequence of the damming, approximately 150 km of river, formerly part of the Middle Paraná River basin, became physically and hydrologically connected to the Upper Paraná River basin (Agostinho & Júlio Júnior, 1999; Dagosta et al., 2024; Graça & Pavanelli, 2007; Júlio Júnior et al., 2009; Langeani et al., 2007; Ota et al., 2018). Considering this reconfiguration, authors like Abell et al. (2008) included the entire stretch of the Paraná River between the former Sete Quedas Falls and the Itaipu Dam within the Upper Paraná ecoregion, which encompasses the Upper Paraná River basin and the artificially incorporated section. Conversely, the Lower Paraná ecoregion is defined as comprising the portion of the Paraná River downstream from the Itaipu Dam, the Iguassu River below the Iguassu Falls, the Paraguay River below the confluence of the Bermejo River and several drainages that flow directly into the Rio de la Plata [Abell et al., 2008; Word Wildlife Fund (WWF), The Nature Conservancy (TNC), 2019]. In this study, we adopt the ecoregional framework of Abell et al. (2008). However, for hydrographic references, we distinguish between the Upper Paraná River basin (above Sete Quedas Falls) and the Middle Paraná River basin (between Sete Quedas and Apipé Falls) based on their historical and biogeographic definitions.

The filling of the Itaipu Reservoir facilitated the access of fish species native to the Middle Paraná River basin into the Upper Paraná River basin. Following this event, numerous species successfully established themselves in this region (Júlio Júnior et al., 2009) and have dispersed extensively, with their movement only constrained by the numerous hydroelectric power stations situated upstream from the Itaipu. The ongoing functionality of the Canal da Piracema (Spawning Channel), established in December 2002, has further contributed to this ichthyofaunal mixing. This channel enables the movement of fish species from the Lower to the Upper Paraná ecoregions surpassing the Itaipu Dam (Agostinho et al., 2015; Makrakis et al., 2007) and theoretically allows the reverse migration of species from the Upper to the Lower Paraná ecoregion. Consequently, closely related species that were historically isolated have had the opportunity to interact. Moreover, instances of dominance have been observed among similar species that were previously isolated by the Sete Quedas (e.g., Ganassin et al., 2021). When these species came to inhabit the newly modified habitats, they demonstrated enhanced reproductive success compared to their native congeners. Over time, the ichthyofauna within the influence zone of the Itaipu Reservoir has undergone significant and likely irreversible changes in species composition and population densities not only in the area flooded by the reservoir but also upstream within the Upper Paraná River basin and potentially downstream in the Lower Paraná ecoregion, affecting both historically distinct faunal assemblages. The systematic documentation of these changes is crucial for comprehending this ongoing ecological transformation. This research should also extend to the tributaries within the inundated area, which, in theory, may still support species in their upstream habitats that have not been adversely affected by the dam's construction, thereby preserving the ecological integrity of their natural flow.

The São Francisco Falso Braço Norte (hereafter, SFFBN) River basin is the second‐largest basin in terms of the drainage area on the east margin of the Itaipu Reservoir (Fernandez & Baller, 2018). This basin belongs to the contemporary Upper Paraná ecoregion (sensu Abell et al., 2008). However, historically, it is part of the Middle Paraná River basin (sensu Říčan et al., 2019). For this reason, the SFFBN River basin is even more relevant for ichthyofaunistic studies, mainly its higher parts, which are little affected by the formation of the Itaipu Reservoir. However, previous research on the ichthyofauna of the SFFBN River basin concentrated sampling points mainly close to the Itaipu Reservoir (see fig. 1b in Reis et al., 2020), besides four streams sampled twice each by Pereira et al. (2021). Additionally, there are still many taxonomic uncertainties and possibly more than a dozen new species revealed by genetic analyses (see Pereira et al., 2021). Furthermore, some studies conducted in the Itaipu Reservoir and its tributaries focused on medium and large species because of the selectivity of the capture methods used (e.g., Benedito‐Cecilio & Agostinho, 2000; Benedito‐Cecílio et al., 1997; Oliveira et al., 2003, 2004, 2005).

Therefore, given the strategic position of the SFFBN River basin, at the intersection of two different basins with endemic fish species that have been artificially placed in permanent contact, taxonomic uncertainties, scarce sampling in the upper reaches of the basin and the selectivity of capture methods in some previous research, this study aimed to carry out an ichthyofaunal inventory alongside the SFFBN River, considering the main channel and its tributaries. Comments on the ichthyofaunal geographic origin, as well as a comprehensive list of species with conservation status and pictures, are provided.

## 2 METHODS

### 2.1 Study area

**FIGURE 1:**
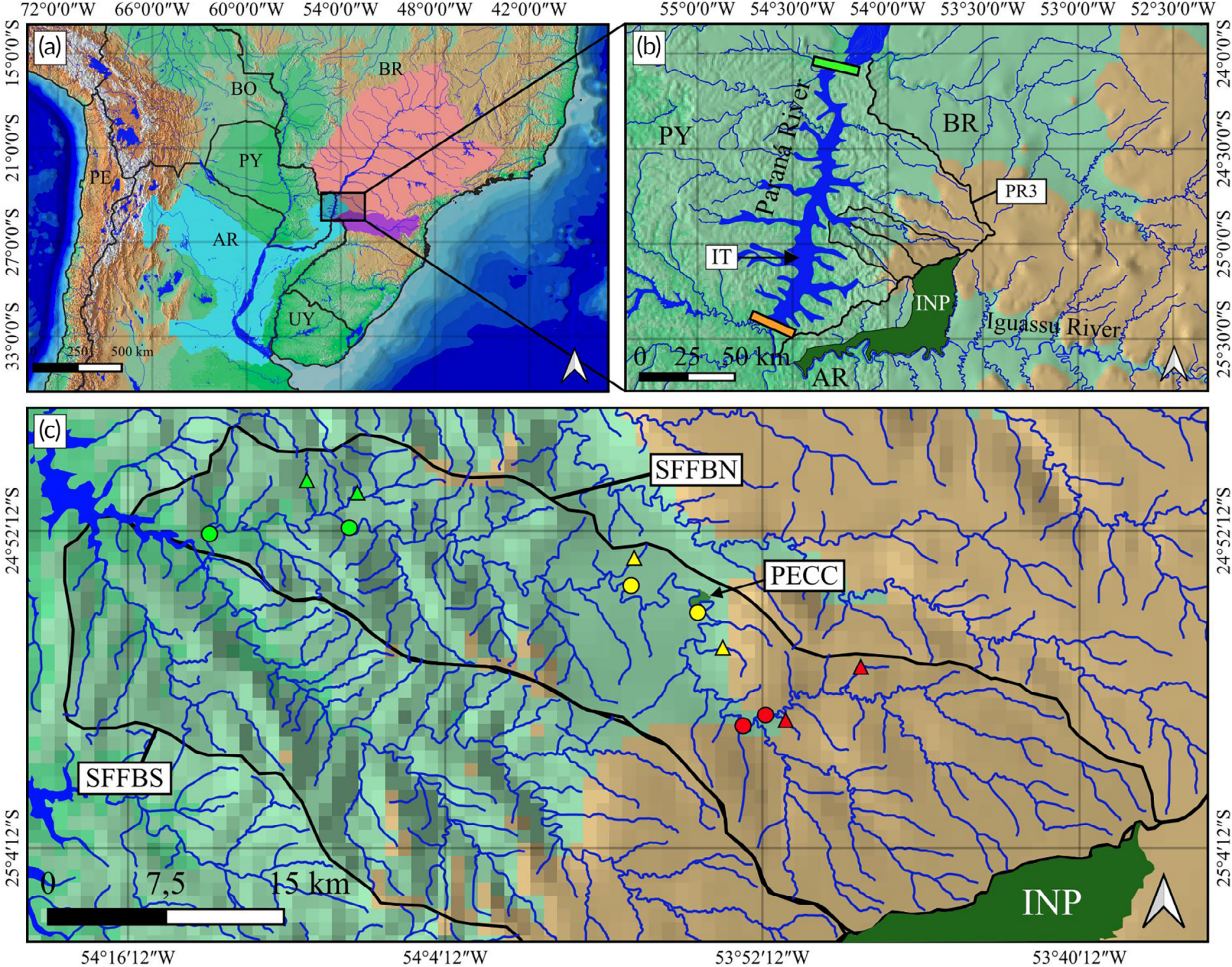
Location of the São Francisco Falso Braço Norte River basin. (a) Lower Paraná (cyan), Iguassu (purple) and Upper Paraná (salmon) ecoregions following Abell et al. (2008) highlighted in part of South America. (b) Location of the Paraná Hydrographic Unit 3 (PR3), Itaipu Reservoir (IT), the protected area Iguassu National Park (INP), Sete Quedas Falls (green bar) and Itaipu Dam (orange bar). (c) São Francisco Falso River Basin Braço Norte (SFFBN) and Braço Sul (SFFBS), with emphasis on the protected area Parque Estadual Cabeça do Cachorro (PECC) and sampling sites on the main channel (circles) and tributaries (triangles) in the lower (green symbols), middle (yellow symbols) and upper (red symbols) altitude region of the basin.

The Paraná Hydrographic Unit 3, also called the Paraná 3 basin [Rocha & Bade, 2018; Instituto Água e Terra (IAT), 2020], has a total area of 8389 km^2^ (Pereira & Scroccaro, 2013) and is in the west of Paraná State, Brazil (Figure 1a). It is delimited to the south by the Itaipu Dam in the Municipality of Foz do Iguaçu and to the north by the Sete Quedas Falls (currently flooded by the Itaipu Reservoir) in the Municipality of Guaíra [Cunha, 2018; Instituto Água e Terra (IAT), 2020] (Figure 1b). After the Itaipu Reservoir was filled, the water dynamics of the Paraná River and its tributaries changed in their lower‐altitude portions immediately above the dam (Fernandez & Baller, 2018). This led to a reconfiguration in the São Francisco Falso River basin, which was split into two independent basins that flow towards the reservoir: SFFBN and Braço Sul (Fernandez & Baller, 2018) (Figure 1b,c).

The SFFBN River basin covers a length of 126.10 km, encompassing an area of 815.07 km^2^, and includes seven municipalities (Fernandez & Baller, 2018). This river originates on the border between the municipalities of Santa Teresa do Oeste and Céu Azul, at an altitude of 718 m a.s.l., and flows into the Itaipu Reservoir in the Municipality of Santa Helena, at an altitude of 220 m a.s.l., with a level difference of 498 m and a declivity of 0.0039 m (Fernandez & Baller, 2018).

### 2.2 Ichthyofauna sampling

**FIGURE 2:**
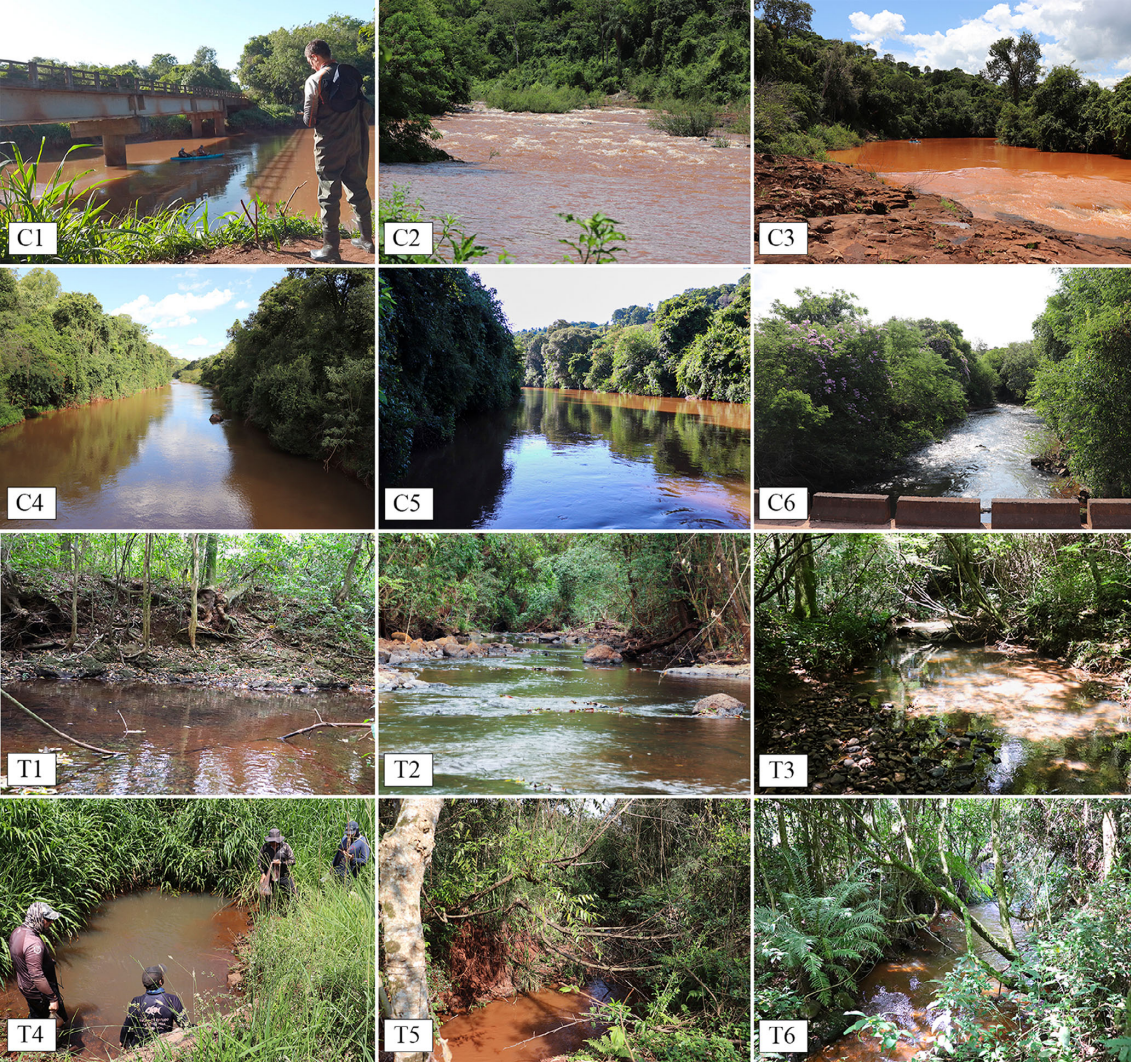
Sampling sites in the São Francisco Falso Braço Norte River basin. Detailed descriptions of the sites in the main channel (C1–C6) and tributaries (T1–T6) are provided in Table 1.

The ichthyofauna was sampled quarterly between August 2022 and May 2023 at 12 sampling sites (Table 1; Figure 2). The SFFBN River basin is not navigable in many sections; therefore, the collection points were first defined according to the roads that allowed access to the sites by land. Sampling sites were distributed along the longitudinal axis of the basin to include two locations in the main channel and two locations on tributaries in each of the lower, middle and upper altitude regions (Figure 1c). All tributaries sampled are first order, except for the Do Ouro stream (T2) in the lower region of the basin, which is third order (Christofoletti, 1980).

**TABLE 1:** Description of sampling sites in the São Francisco Falso Braço Norte (SFFBN) River basin.

Site	Co‐ordinates	Altitude (m)	Description
C1	24°52′4″ S 54°13′11″ W	228	SFFBN River, close to its mouth at Itaipu Reservoir; lotic environment, medium flow; turbid water; riparian vegetation present; substrate on the banks composed of earth.
C2	24°52′5″ S 54° 7′50″ W	249	SFFBN River; lotic environment, containing stretches of rapids; turbid water; riparian vegetation present; substrate composed of rocks, gravel and earth in some sections.
C3	24°54′16″ S 53°57′6″ W	376	SFFBN River; lotic environment, containing stretches of rapids; turbid water; riparian vegetation present; substrate composed of rocks and gravel.
C4	24°55′17″ S 53°54′26″ W	396	SFFBN River; lotic environment; turbid water; the protected area Parque Estadual Cabeça do Cachorro is located on the right bank; substrate composed of rocks and gravel.
C5	24°59′38″ S 53°52′55″ W	440	SFFBN River; lotic environment; turbid water; riparian vegetation present; substrate composed of rocks and gravel.
C6	24°59′16″ S 53°52′25″ W	446	SFFBN River; lotic environment; turbid water; riparian vegetation present; substrate composed of rocks and gravel.
T1	24°50′31″ S 54°9′24″ W	268	Barreirinho stream; lotic environment; clear water; riparian vegetation present; substrate composed of rocks, gravel and plant litter.
T2	24°50′48″ S 54°7′35″ W	250	Do Ouro stream; lotic environment; clear water; riparian vegetation present; substrate composed of rocks, gravel and plant litter; moss present on several rocks.
T3	24°53′23″ S 53°57′9″ W	406	Stream with unknown name; lotic environment; clear water; riparian vegetation present; substrate composed of rocks, gravel and plant litter.
T4	24°56′36″ S 53°53′36″ W	485	Campina Grande stream; lotic environment; the water is turbid and forms a puddle with abundant grasses upstream of the road; riparian vegetation is present; substrate composed of rocks and gravel.
T5	24°59′13″ S 53°51′31″ W	453	Stream with unknown name; lotic environment; clear water; riparian vegetation present; substrate composed of rocks and gravel.
T6	24°57′38″ S 53°48′39″ W	516	Stream with unknown name; lotic environment; clear and whitish water; water spring area, riparian vegetation present; substrate composed of rocks, gravel and clay.

*Note*: Sites on the main channel (C1–C6) and tributaries (T1–T6).

Fish were collected using active and passive methods. The passive method consisted of using gillnets (30–70 mm mesh) exposed for approximately 12 h. The active method consisted of employing a cast‐net (10.0 mm mesh), sieves (87 × 65 cm and 115 × 85 cm; 1.0 mm mesh) and a seine net (10 m long and 2.4 mm mesh). The active method was used at all sampling points. In contrast, the passive method was used only in the main channel due to the small size of the tributaries, which made the use of the set of gillnets unfeasible. Samplings were authorized by the Sistema de Autorização e Informação em Biodiversidade (SISBIO), licence no. 81,459, and by the Comissão de Ética no Uso de Animais (CEUA) of the Universidade Tecnológica Federal do Paraná (UTFPR), process no. 2022–01.

After capture, fishes were killed in 3000 mg/L eugenol (Lucena et al., 2013), fixed in 10% formaldehyde and preserved in 70° GL alcohol (Auricchio & Salomão, 2002). Species were identified using identification guides (e.g., Britski et al., 2007; Baumgartner et al., 2012; Almirón et al., 2015; Ota et al., 2018; Gimênes‐Júnior & Rech, 2022), other specialized literature and original descriptions [e.g., Kullander (1982), Varella (2011), and Varella et al. (2023) for *Saxatilia* species; Kullander (2009), Lucena et al. (2013), Varella (2011), and Říčan et al. (2023) for *Crenicichla* species; Azpelicueta et al. (2002), Miquelarena and Menni (2005), Melo and Buckup (2006), Bertaco and Lucena (2006), Vari and Castro (2007), Garavello and Sampaio (2010), Lucena and Soares (2016), Oliveira (2017), and Terán et al. (2020) for *Astyanax* and *Psalidodon* species], as well as comparison with specimens deposited in the Coleção Ictiológica da Universidade Tecnológica Federal do Paraná, *Campus* Santa Helena, Paraná State, Brazil (CISH), and the Coleção Ictiológica do Núcleo de Pesquisas em Limnologia, Ictiologia e Aquicultura, Universidade Estadual de Maringá, Paraná State, Brazil (NUP). Data on the conservation status follow International Union for Conservation of Nature (IUCN) (2023) as the study area is near the tri‐border between Brazil, Argentina and Paraguay. When the species was not evaluated by the IUCN, data from Instituto Chico Mendes de Conservação da Biodiversidade (ICMBio) (2024), which refers exclusively to Brazilian territory, were used. The conservation status of *Satanoperca setepele* followed Ota et al. (2021).

Determining native species in the study area is quite challenging due to the mixture of fauna between the Middle and Upper Paraná River basins. Therefore, information regarding the geographic distribution of species in relation to their natural distribution above and below the former Sete Quedas Falls was compiled from the works of Agostinho and Júlio Júnior (1999), Júlio Júnior et al. (2009), Ota et al. (2018), Reis et al. (2020), Fricke et al. (2023), Pavanelli et al. (2023) and Dagosta et al. (2024). Seven categories were established to classify the geographic distribution of the species: (A) natural distribution in the Paraná River basin both above and below the Sete Quedas Falls; (B) natural distribution in the Paraná River basin just below the Sete Quedas Falls; (C) natural distribution in the Paraná River basin only above the Sete Quedas Falls; (D) previously endemic to the Iguassu River basin; (E) native to other Neotropical regions and non‐native in the Paraná River basin; (F) native to another continent; or (G) of uncertain origin in the study area.

### 2.3 Data analysis

To assess sampling efficiency, a species accumulation curve was generated using the bootstrap method (Smith & Van Belle, 1984) along with the standard error, utilizing the ‘specaccum’ function from the ‘vegan’ package (Oksanen et al., 2014) in R version 4.0 (R Development Core Team, 2019).

Each sampled site was treated as a sampling unit. The abundance of individuals and species richness (*S*) were calculated by the sum of the four capture events in each sampled site. Shannon diversity index (*H′*) (Shannon, 1948) and Pielou evenness index (E) (Pielou, 1966) were used to verify the heterogeneity among the sampling points. These analyses were performed separately for each sampling point and for the basin's lower, middle and upper regions by integrating the data from all sampling points in the same altimetric region. Differences in species composition among the sampling points and altimetric regions were verified using the Bray–Curtis similarity index (Bray & Curtis, 1957). These analyses were performed in the PAST software, version 4.03 (Hammer et al., 2001).

## 3 RESULTS

**FIGURE 3:**
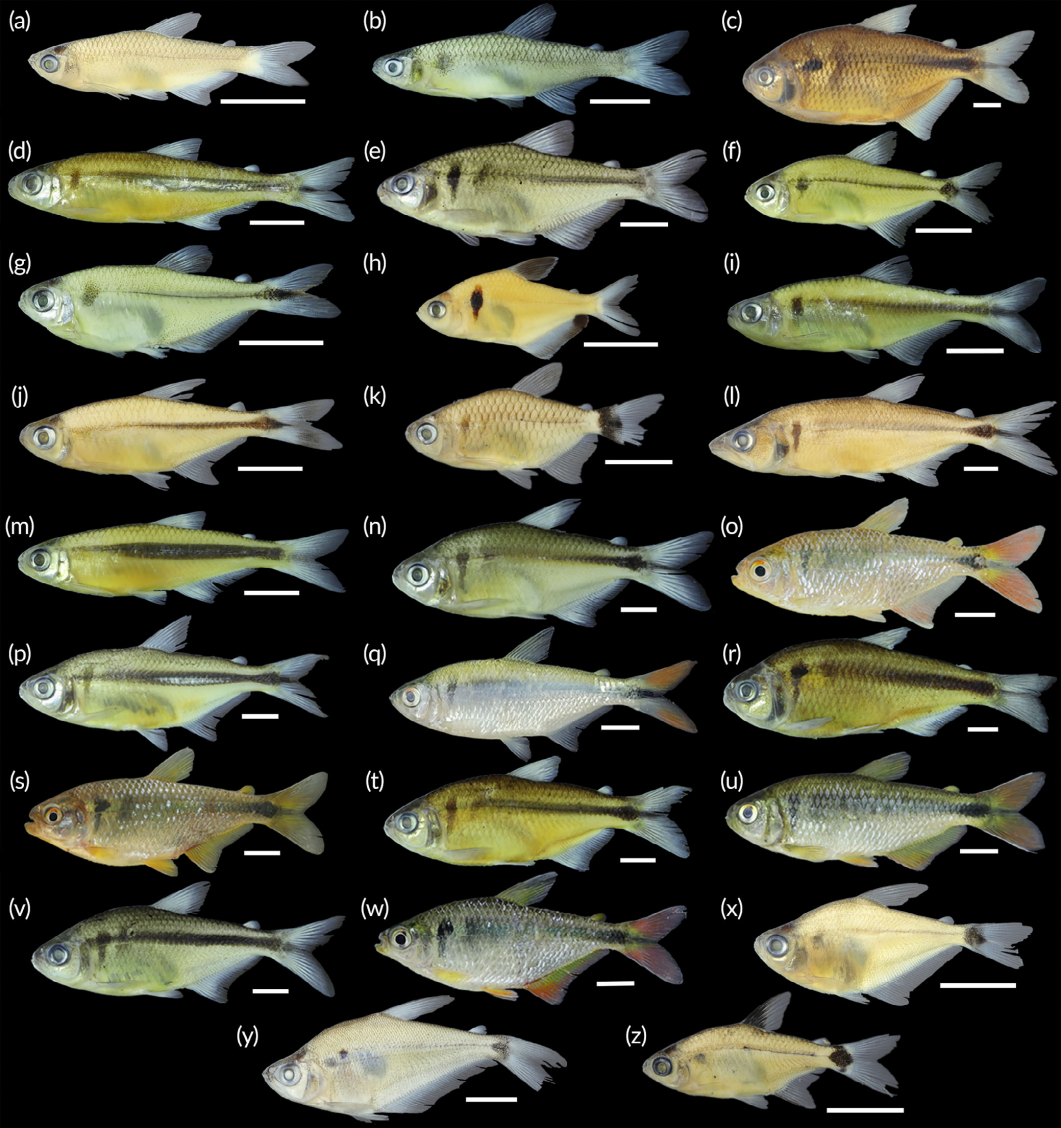
Acestrorhamphidae, Characidae and Stevardiidae from the São Francisco Falso Braço Norte River basin. Colour in alcohol, except when mentioned. (a) *Aphyocharax anisitsi*, CISH 734; (b) *Aphyocharax* sp., NUP 24635; (c) *Astyanax lacustris*, CISH 750; (d) *Bryconamericus exodon*, NUP 24600; (e) *Bryconamericus ikaa*, NUP 24632; (f) *Deuterodon luetkenii*, NUP 24631; (g) *Diapoma guarani*, NUP 24615; (h) *Megalamphodus eques*, CISH 238; (i) *Knodus* aff. *moenkhausii*, NUP 24617; (j) *Moenkhausia bonita*, CISH 740; (k) *Bario forestii*, CISH 730; (l) *Oligosarcus paranensis*, CISH 743; (m) *Piabarchus stramineus*, NUP 24602. (n) *Psalidodon bockmanni*, NUP 24629; (o) *P. bockmanni*, NUP 24629, colour in life; (p) *Psalidodon* aff. *fasciatus*, CISH 821; (q) *P*. aff. *fasciatus*, CISH 821, colour in life; (r) *Psalidodon troya*, NUP 24612; (s) *P. troya*, CISH 870, colour in life; (t) *Psalidodon* sp. ‘false‐paranae’, NUP 24593; (u) *Psalidodon* sp. ‘false‐paranae’, CISH 872, colour in life; (v) *Psalidodon* sp. ‘striped’, NUP 24634; (w) *Psalidodon* sp. ‘striped’, CISH 863, colour in life; (x) *Ctenobrycon kennedyi*, CISH 744; (y) *Roeboides descalvadensis*, CISH 745; (z) *Serrapinnus notomelas*, CISH 746. Scale bars = 10 mm.

**FIGURE 4:**
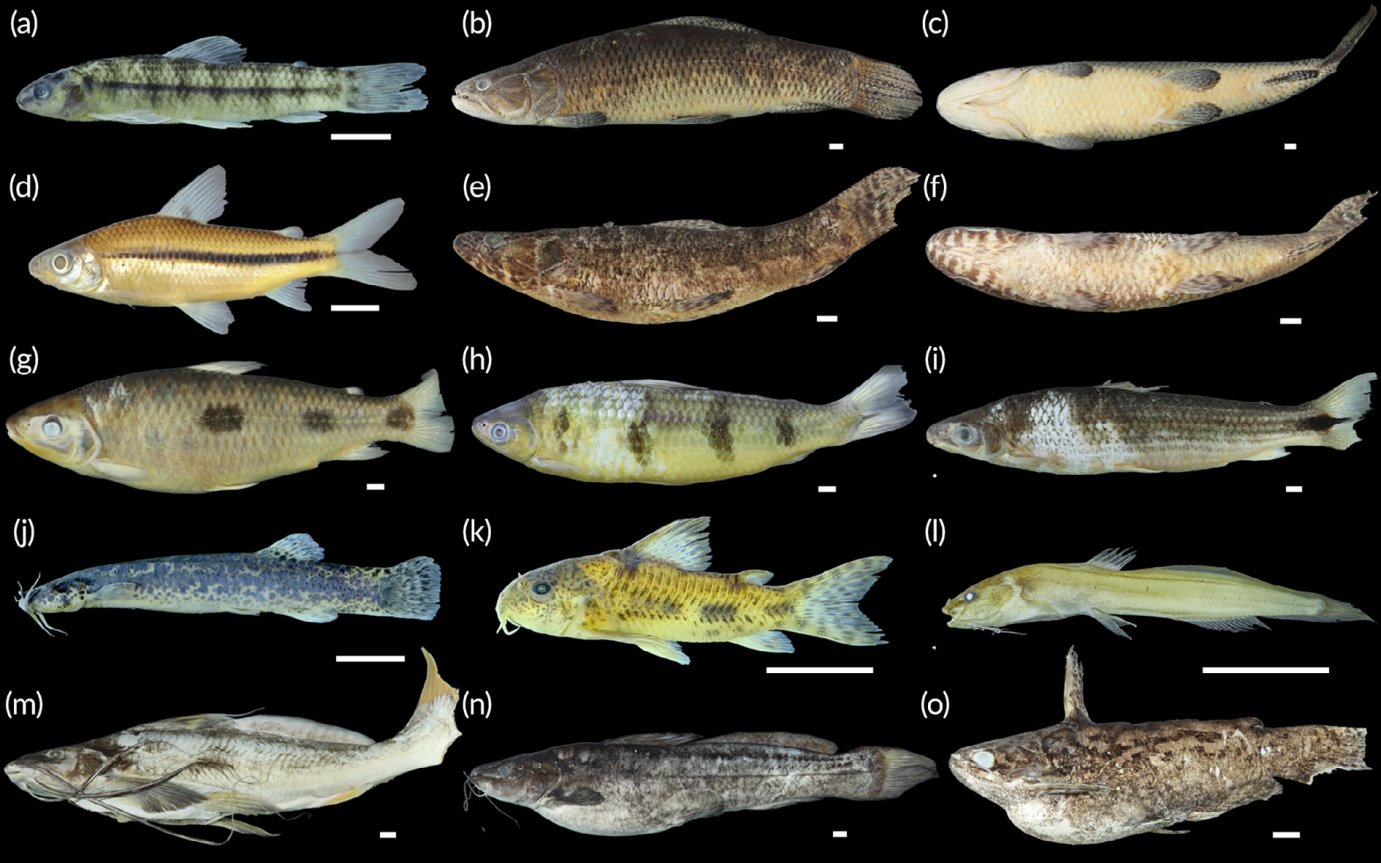
Other Characiformes and Siluriformes (non‐Loricariidae) from the São Francisco Falso Braço Norte River basin. (a) *Characidium* aff. *zebra*, NUP 24630; (b) *Hoplias* aff. *malabaricus*, CISH 694; (c) *H*. aff. *malabaricus*, CISH 694, ventral view; (d) *Steindachnerina brevipinna*, CISH 748; (e) *Hoplias mbigua*, CISH 700; (f) *H. mbigua*, CISH 700, ventral view; (g) *Leporinus friderici*, CISH 688; (h) *Schizodon borellii*, NUP 24616; (i) *Schizodon nasutus*, CISH 696; (j) *Cambeva* aff. *davisi*, NUP 24622; (k) *Hoplisoma* cf. *carlae*, NUP 24595; (l) *Heptapterus mustelinus*, NUP 24614; (m) *Pinirampus pirinampu*, CISH 706; (n) *Rhamdia* aff. q*uelen*, CISH 693; (o) *Trachelyopterus galeatus*, CISH 815. Scale bars = 10 mm.

**FIGURE 5:**
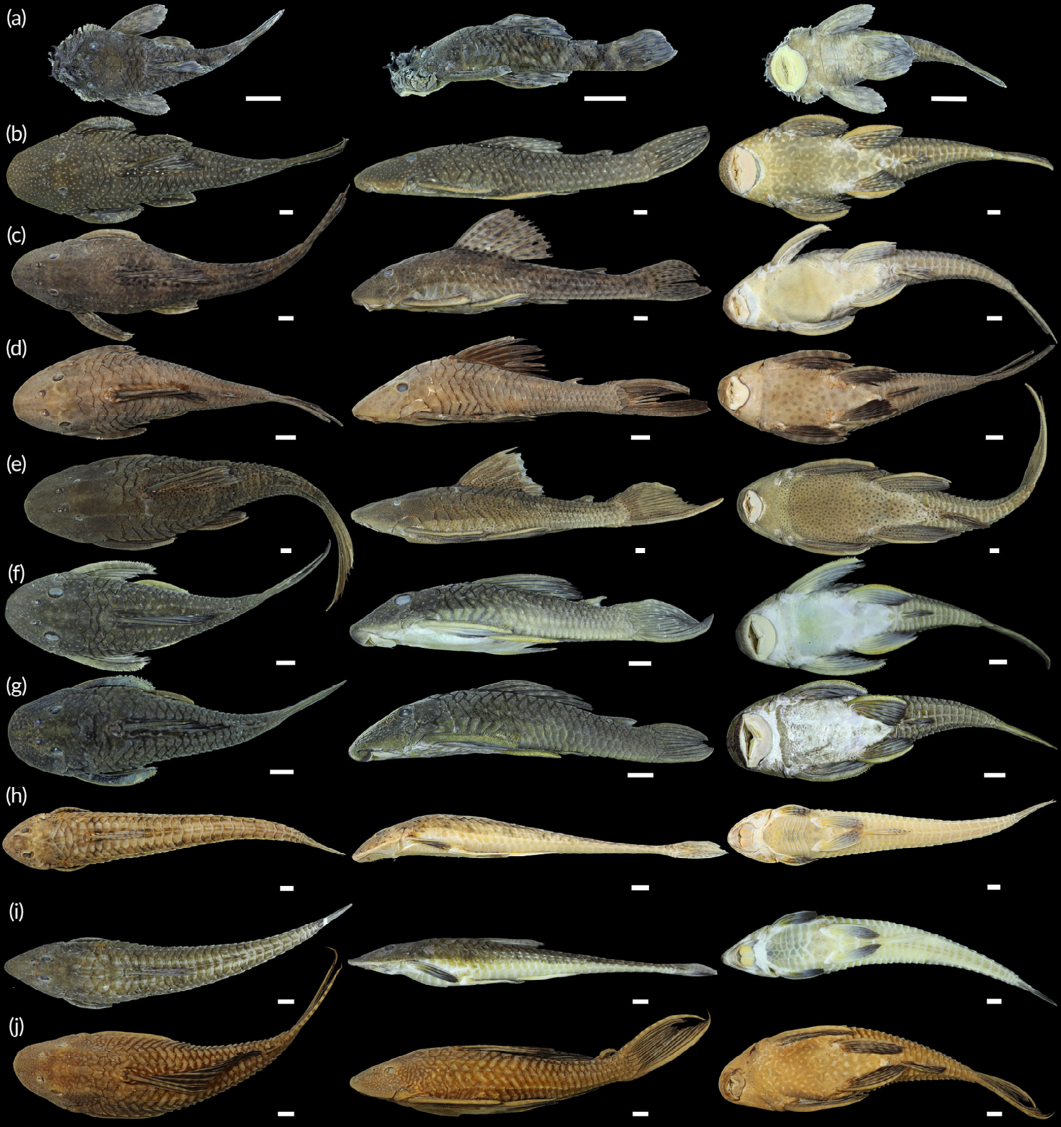
Loricariidae from the São Francisco Falso Braço Norte River basin. Dorsal (left), lateral (central) and ventral (right) views: (a) *Ancistrus* sp., NUP 24613; (b) *Hypostomus albopunctatus*, CISH 680; (c) *Hypostomus ancistroides*, CISH 809; (d) *Hypostomus cochliodon*, CISH 812; (e) *Hypostomus commersoni*, CISH 703; (f) *Hypostomus strigaticeps*, NUP 24601; (g) *Hypostomus* aff. *tietensis*, NUP 24596; (h) *Loricariichthys platymetopon*, CISH 707; (i) *Loricariichthys rostratus*, NUP 24620; (j) *Pterygoplichthys ambrosettii*, CISH 452. Scale bars = 10 mm.

**FIGURE 6:**
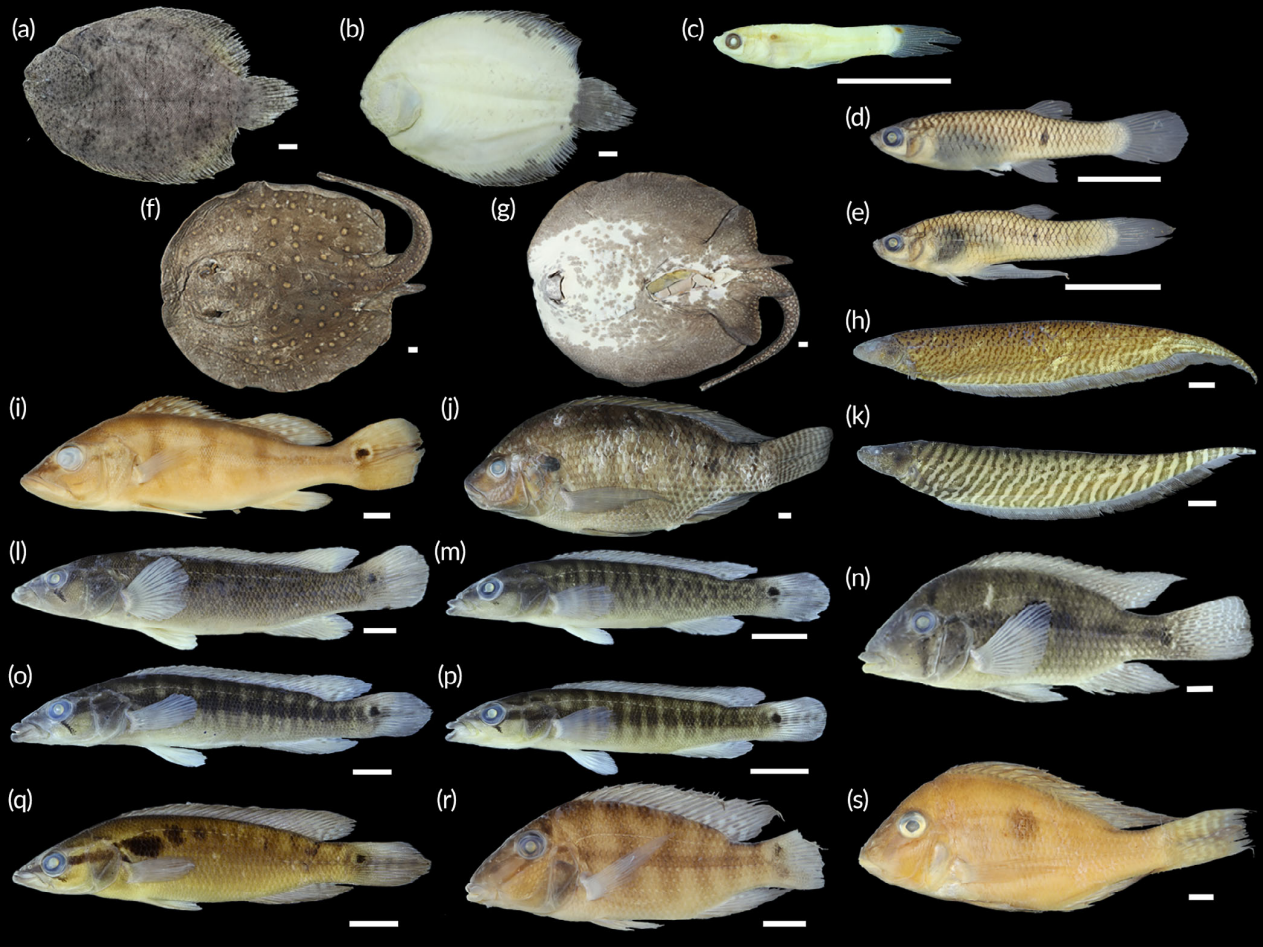
Carangiformes, Cichliformes, Cyprinodontiformes, Myliobatiformes and Gymnotiformes from the São Francisco Falso Braço Norte River basin. (a) *Catathyridium jenynsii*, NUP 24637, left view; (b) *C. jenynsii*, NUP 24637, right view; (c) *Poecilia reticulata*, CISH 784, male; (d) *Phalloceros harpagos*, CISH 783, female; (e) *P. harpagos*, CISH 783, male; (f) *Potamotrygon amandae*, CISH 697, dorsal view; (g) *P. amandae*, CISH 697, ventral view; (h) *Gymnotus* aff. *inaequilabiatus*, NUP 24606; (i) *Cichla kelberi*, CISH 114; (j) *Oreochromis niloticus*, CISH 692; (k) *Gymnotus* aff. *sylvius*, NUP 24628; (l) *Creniciclha* cf. *gillmorlisi*, NUP 24639, adult; (m) *C*. cf. *gillmorlisi*, NUP 24639, juvenile; (n) ‘*Geophagus*’ *iporanguensis*, NUP 24604; (o) *Crenicichla mandelburgueri*, NUP 24636, adult; (p) *C. mandelburgueri*, NUP 24636, juvenile; (q) *Saxatillia britskii*, NUP 24590; (r) *Satanoperca setepele*, CISH 764; (s) *Geophagus sveni*, CISH 859. Scale bars = 10 mm.

**FIGURE 7:**
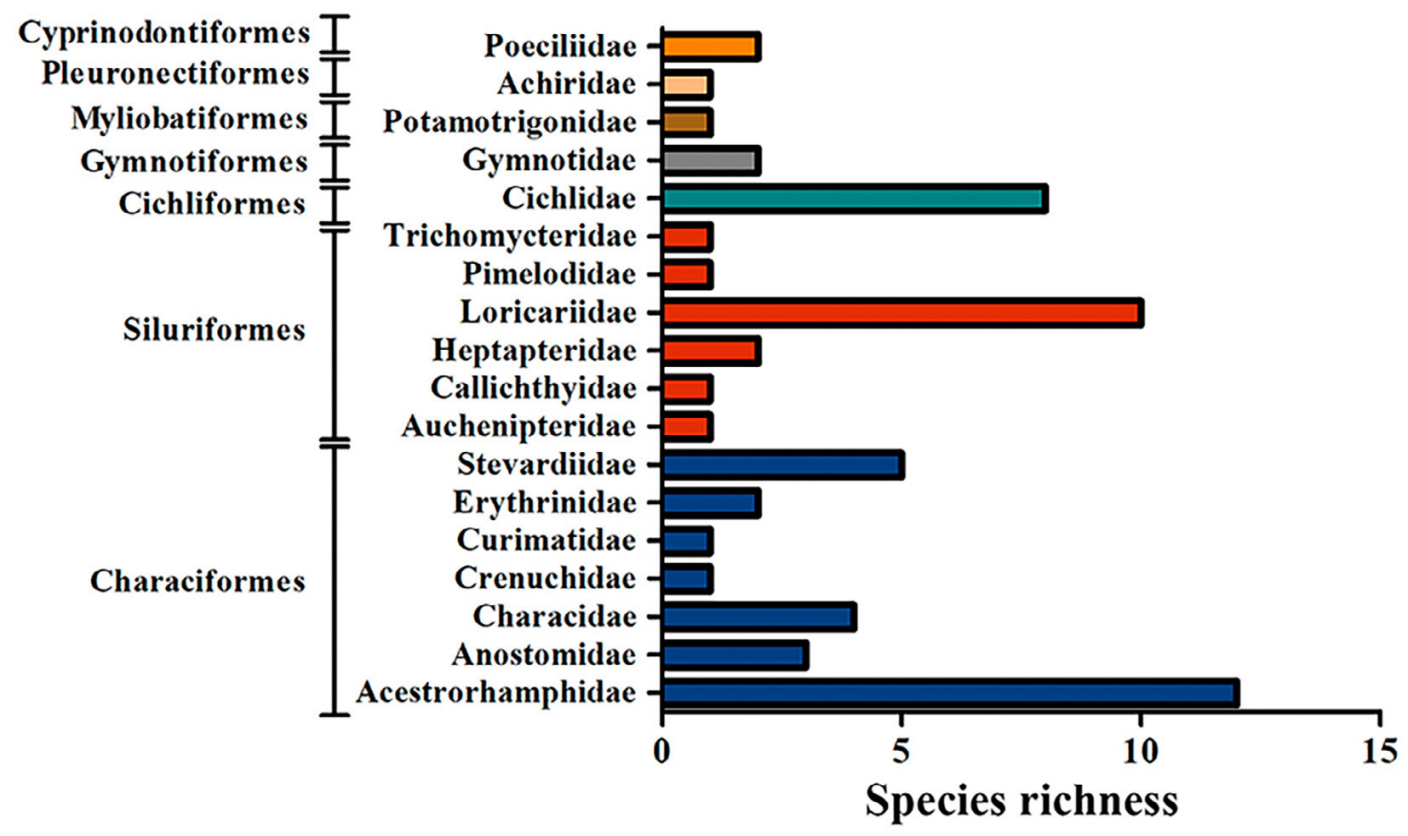
Species richness by order and family sampled in the São Francisco Falso Braço Norte River basin. Families within the same order are represented by the same colour.

**FIGURE 8:**
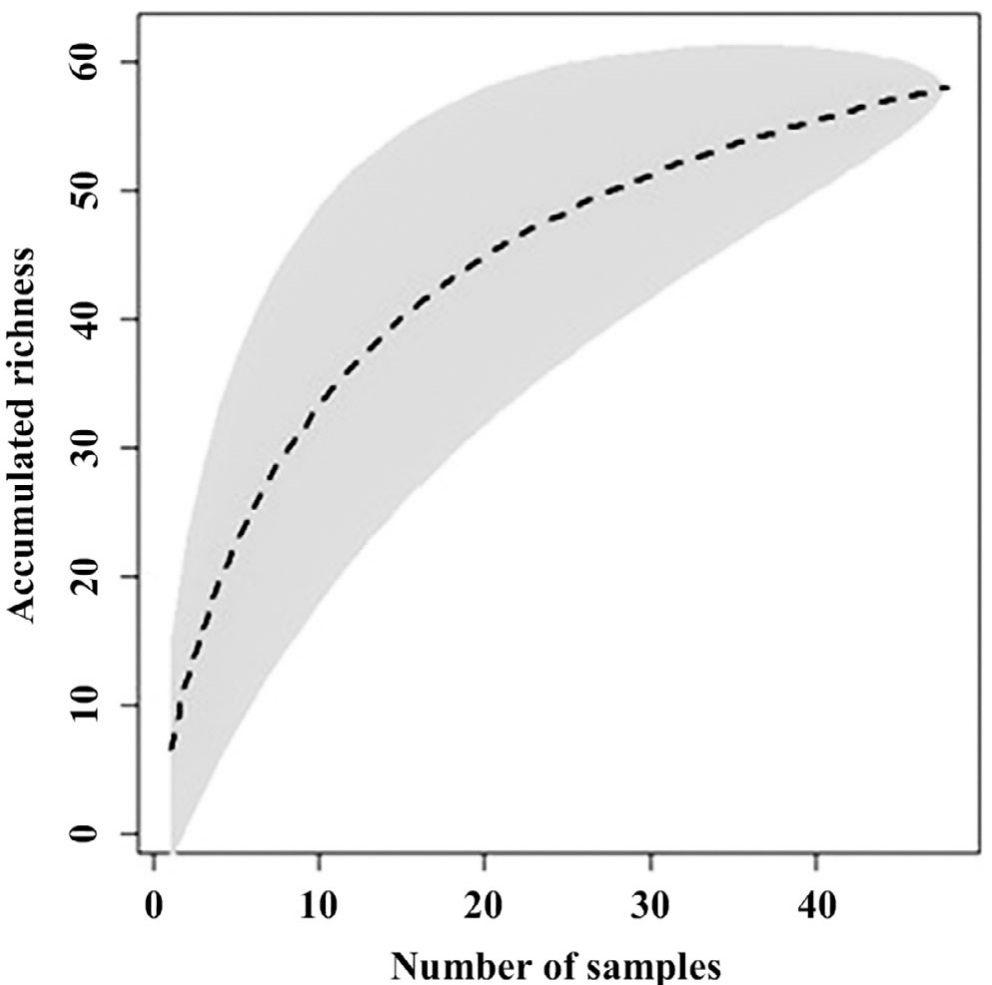
Cumulative curve of the number of species captured in the São Francisco Falso Braço Norte River basin based on the sampling effort.

A total of 1844 fish were collected, representing two classes, seven orders, 18 families, 41 genera and 58 species (Table 2; Figures 3, 4, 5, 6). Characiformes and Siluriformes exhibited the highest diversity, comprising 28 (48.3%) and 16 (27.6%) species, respectively (Figure 7). These two orders accounted for 86.2% of all fish captured. The families Acestrorhamphidae, Loricariidae and Cichlidae were the most diverse, with 12 (20.7%), 10 (17.2%) and 8 (13.8%) species, respectively (Figure 7). Acestrorhamphidae, Stevardiidae and Loricariidae represented most of the catch, comprising 71.9% of the total fish collected. The cumulative species curve did not reach an asymptote (Figure 8), and the estimated total richness was 64 species.

**TABLE 2:** Fish species collected in the São Francisco Falso Braço Norte River basin.

Classification	*N*	Origin/cons.	Sampling sites												Voucher
			Main channel						Tributaries						
			C1	C2	C3	C4	C5	C6	T1	T2	T3	T4	T5	T6	
ELASMOBRANCHII															
MYLIOBATIFORMES															
Potamotrygonidae															
1. *Potamotrygon amandae* Loboda & Carvalho, 2013	3	B/LC	X												CISH 697
ACTINOPTERI															
GYMNOTIFORMES															
Gymnotidae															
2. *Gymnotus* aff. *inaequilabiatus* (Valenciennes, 1839)	2	G	X					X							NUP 24606; CISH 689
3. *Gymnotus* aff. *sylvius* Albert & Fernandes‐Matioli, 1999	8	G			X		X					X			NUP 24628
CHARACIFORMES															
Crenuchidae															
4. *Characidium* aff. *zebra* Eigenmann, 1909	60	A							X	X	X	X		X	NUP 24610; CISH 724
Erythrinidae															
5. *Hoplias* aff. *malabaricus* (Bloch, 1794)	6	C				X	X	X							NUP 24641; CISH 694
6. *Hoplias mbigua* Azpelicueta, Benítez, Aichino & Mendez, 2015	14	B	X	X		X		X							NUP 24638; CISH 700
Anostomidae															
7. *Leporinus friderici* (Bloch, 1794)^LDM^	23	A/LC	X	X											NUP 24619; CISH 688
8. *Schizodon borellii* (Boulenger, 1900)^LDM^	2	B/LC	X												NUP 24591; CISH 696
9. *Schizodon nasutus* Kner, 1858^LDM^	2	C/LC	X												NUP 24616
Curimatidae															
10. *Steindachnerina brevipinna* (Eigenmann & Eigenmann, 1889)	17	B	X	X											CISH 747
Stevardiidae															
Diapominae															
11. *Bryconamericus exodon* Eigenmann, 1907	39	B/LC		X											NUP 24600; CISH 729
12. *Bryconamericus ikaa* Casciotta, Almirón & Azpelicueta, 2004^N^	93	D/LC			X	X	X	X	X	X	X		X		NUP 24594, 24,605
13. *Diapoma guarani* (Mahnert & Géry, 1987)	53	B/LC	X												NUP 24615
14. *Knodus* aff. *moenkhausii* (Eigenmann & Kennedy, 1903)	24	B	X						X	X					NUP 24617; CISH 730
15. *Piabarchus stramineus* (Eigenmann, 1908)	69	A/LC	X	X						X					NUP 24602
Characidae															
Aphyocharacinae															
16. *Aphyocharax anisitsi* Eigenmann & Kennedy, 1903	9	B/LC	X												CISH 734
17. *Aphyocharax* sp.	51	A	X	X											NUP 24635; CISH 735
Characinae															
18. *Roeboides descalvadensis* Fowler, 1932	4	B/LC	X												CISH 745
Cheirodontinae															
19. *Serrapinnus notomelas* (Eigenmann, 1915)	28	A/LC	X	X	X		X						X		CISH 746
Acestrorhamphidae															
Stygichthyinae															
20. *Deuterodon luetkenii* (Boulenger, 1887)	9	B/LC	X												NUP 24631; CISH 733
Megalamphodinae															
21. *Megalamphodus eques* (Steindachner, 1882)	1	G/LC	X												CISH 736
Stichonodontinae															
22. *Moenkhausia bonita* Benine, Castro & Sabino, 2004	58	A/LC	X	X											CISH 740
Thayeriinae															
23. *Bario forestii* (Benine, Mariguela & Oliveira, 2009)	6	B/LC	X												CISH 742
Acestrorhamphinae															
24. *Astyanax lacustris* (Lütken, 1875)	154	A/LC	X	X	X	X	X	X	X	X	X	X	X		CISH 750
25. *Ctenobrycon kennedyi* (Eigenmann, 1903)	10	B/LC	X												CISH 744
26. *Oligosarcus paranensis* Menezes & Géry, 1983	25	A/LC			X		X	X		X	X				NUP 24640; CISH 743
27. *Psalidodon bockmanni* (Vari & Castro, 2007)	36	C/LC										X			NUP 24629; CISH 822
28. *Psalidodon* aff. *fasciatus* (Cuvier, 1819)	1	A		X											CISH 821
29. *Psalidodon troya* (Azpelicueta, Casciotta & Almirón, 2002)^N^	382	B/VU			X	X	X	X		X	X	X	X	X	NUP 24612, 24,607; CISH 855
30. *Psalidodon* sp. ‘false‐paranae’	165	B			X	X	X	X	X	X	X		X		NUP 24593; CISH 836
31. *Psalidodon* sp. ‘striped’	23	B	X						X	X					NUP 24592; CISH 863
SILURIFORMES															
Trichomycteridae															
32. *Cambeva* aff. *davisi* (Haseman, 1911)	24	A					X				X	X			NUP 24622; CISH 788
Callichthyidae															
33. *Hoplisoma* cf. *carlae* (Nijssen & Isbrücker, 1983)^N^	1	D/LC							X						NUP 24595
Loricariidae															
Loricariinae															
34. *Loricariichthys platymetopon* Isbrücker & Nijssen, 1979	13	B/LC	X												NUP 24603; CISH 705
35. *Loricariichthys rostratus* Reis & Pereira, 2000	1	B/LC		X											NUP 24620
Hypostominae															
36. *Ancistrus* sp.	32	G				X	X			X	X		X		NUP 24613; CISH 771
37. *Hypostomus albopunctatus* (Regan, 1908)	57	A/LC			X	X		X							CISH 710
38. *Hypostomus ancistroides* (Ihering, 1911)	48	A/LC	X	X	X	X						X			NUP 24597; CISH 805
39. *Hypostomus cochliodon* Kner, 1854	1	B/LC		X											CISH 812
40. *Hypostomus commersoni* Valenciennes, 1836	8	B/LC	X			X	X	X							NUP 24642; CISH 703
41. *Hypostomus strigaticeps* (Regan, 1908)	12	C/LC		X	X										NUP 24601; CISH 811
42. *Hypostomus* aff. *tietensis* (Ihering, 1905)	6	G		X	X	X									NUP 24596; CISH 799
43. *Pterygoplichthys ambrosettii* (Holmberg, 1893)	1	B/LC	X												CISH 683
Auchenipteridae															
44. *Trachelyopterus galeatus* (Linnaeus, 1766)	4	A/LC	X	X											CISH 815
Heptapteridae															
45. *Heptapterus mustelinus* (Valenciennes, 1835)	3	B/LC								X					NUP 24614
46. *Rhamdia* aff. *quelen* (Quoy & Gaimard, 1824)^LDM^	14	A		X	X	X		X	X				X		NUP 24624; CISH 701
Pimelodidae															
47. *Pinirampus pirinampu* (Spix & Agassiz, 1829)^LDM^	1	A/LC	X												CISH 706
CARANGIFORMES															
Achiridae															
48. *Catathyridium jenynsii* (Günther, 1862)	3	B/LC	X												NUP 24637
CYPRINODONTIFORMES															
Poeciliidae															
49. *Poecilia reticulata* Peters, 1859	1	E/LC											X		CISH 784
50. *Phalloceros* aff. *harpagos* Lucinda, 2008	86	A/LC	X		X	X	X				X	X	X		NUP 24611; CISH 783
CICHLIFORMES															
Cichlidae															
51. *Cichla kelberi* Kullander & Ferreira, 2006	1	E/LC	X												CISH 756
52. *Crenicichla* cf. *gillmorlisi* Kullander & Lucena, 2013 ^N^	7	B			X	X		X							NUP 24639; CISH 868
53. *Crenicichla mandelburgeri* Kullander, 2009 ^N^	17	B/NT		X											NUP 24636
54. ‘*Geophagus*’ *iporangensis* Haseman, 1911	97	C/LC		X	X	X	X	X							NUP 24604; CISH 791
55. *Geophagus sveni* Lucinda, Lucena & Assis, 2010	1	E/LC	X												CISH 859
56. *Oreochromis niloticus* (Linnaeus, 1758)	7	F/LC			X				X			X			CISH 692
57. *Satanoperca setepele* Ota, Deprá, Kullander, Graça & Pavanelli, 2022	2	E/LC	X												CISH 764
58. *Saxatilia britskii* (Kullander, 1982)	19	C/LC		X	X	X	X				X				NUP 24590

*Note*: Classification follows Van de Laan et al. (2025). Absolute abundance of individuals (*N*), geographic origin [(a) native to both above and below the Sete Quedas Falls; (b) native to below the Sete Quedas Falls; (c) native to above the Sete Quedas Falls; (d) previously endemic to the Iguaçu Iguassu River basin; (e) non‐native in the Paraná River basin; (f) native to another continent; or (g) of uncertain origin in the study area], conservation status (Cons.), sampling sites (C1–C6 and T1–T6) and vouchers. Conservation status categories according to the International Union for Conservation of Nature (IUCN) (2023): LC, least concern; NT, near threatened; VU, vulnerable. New record for the Paraná River basin above the Itaipu Dam indicated by^N^ and long‐distance migratory species follows Agostinho et al. (2007) by^LDM^.

The active capture method recorded 44 species, 31 of which were exclusive to this method. The passive method recorded 27 species, 14 of which were exclusive to it. Thirteen species were sampled using both capture methods. Focusing solely on the active capture method, the most abundant species were *Psalidodon troya* (23.7%), *Psalidodon* sp. ‘false‐paranae’ (10.3%) and *Astyanax lacustris* (9.5%). In contrast, the most abundant species recorded by the passive capture method were *Hypostomus albopunctatus* (24.4%), *Hypostomus ancistroides* (15.0%) and ‘*Geophagus’ iporangensis* (14.1%).

**FIGURE 9:**
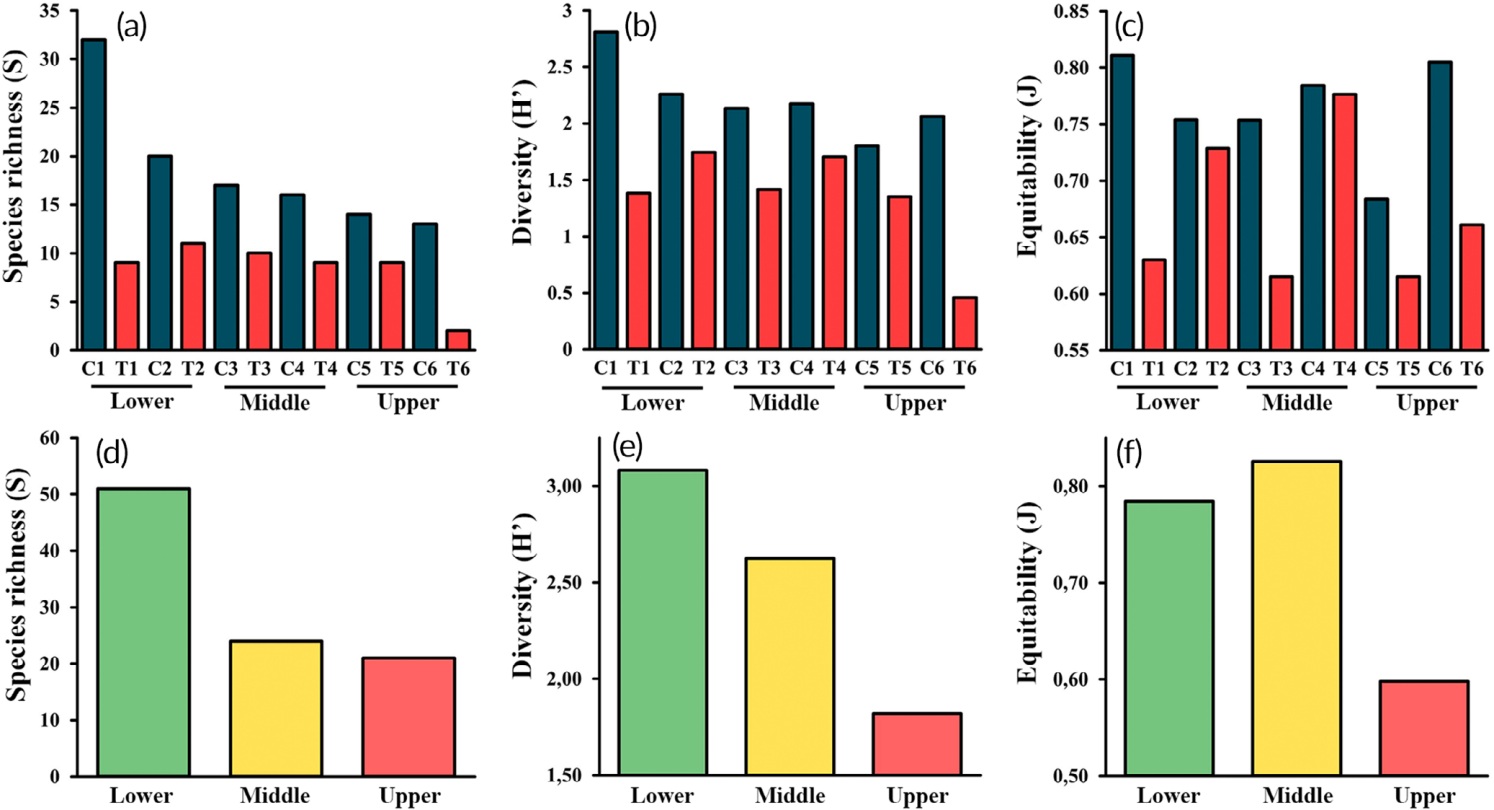
Species richness (a, d), diversity (b, e) and equitability (c, f) at each sampling site (a–c) and altimetric regions (d–f) in the São Francisco Falso Braço Norte River basin. Sampling sites in the main channel (C1–C6) and tributaries (T1–T6) are arranged based on location in the basin's lower, middle and upper regions.

Along the main channel, the highest species richness and diversity were recorded at sampling site C1 (*S* = 31; *H*′ = 2.81) (Figure 9a–c). The most abundant species captured by the active method at this site was *Diapoma guarani* (24.1%), whereas the most abundant species captured by the passive method was *Loricariichthys platymetopon* (25.0%). After this, sampling sites C2 and C3 demonstrated significant richness, with 20 and 17 species, respectively. The most abundant species at these sites, captured by the active method, were *Piabarchus stramineus* (34.3%) and ‘*G*’. *iporangensis* (26.7%), respectively. The most abundant species captured by the passive method in these sites were *Leporinus friderici* (36.8%) and *H. albopunctatus* (54.3%), respectively. The lowest species richness was observed at site C6 (*S* = 13), whereas the lowest diversity was at site C5 (*H*′ = 1.80). Despite the lower species richness, there was outstanding evenness at site C6 (*J* = 0.81), whereas site C5 was dominated mainly by *A. lacustris* (48.3% of captures; *J* = 0.68), resulting in lower diversity values.

Considering only the tributaries, species richness was similar among the sampling sites (Figure 9a–c). The highest richness was at the sampling site T2, with 11 species, followed by sampling site T3, with 10 species. Sampling sites T1, T4 and T5 recorded nine species each. The sampling site T2 was also the most diverse (*H*′ = 1.75) followed by T4 (*H*′ = 1.71). The latter also presented higher values of equitability (*J* = 0.78) followed by T2 (*J* = 0.73). The sampling site T6 was the least rich and diverse (*S* = 2; *H*′ = 0.46). Among the tributaries, the most abundant species were *P. troya* (40.6%), *Psalidodon* sp. ‘false‐paranae’ (17.1%) and *Phalloceros* aff. *harpargos* (8.9%).

Considering the basin's lower, middle and upper regions, there was a longitudinal gradient in richness and diversity, with higher values observed closer to the mouth in Itaipu Reservoir (Figure 9d–e). In contrast, the equitability was higher in the middle stretch (Figure 9f). A total of 51 species (32 exclusive) were recorded in the lower region of the SFFBN River basin (sampling sites C1, C2, T1 and T2), with 42 species identified in the main channel and 14 in the tributaries. In this region, the most abundant species captured using the passive method was *L. friderici* (25.6%). Considering the active method, *P. stramineus* emerged as the most abundant species in the main channel (16.8%), whereas *Psalidodon* sp. ‘false‐paranae’ was predominant in the tributaries (50.4%). In the middle region of the basin (sampling sites C3, C4, T3 and T4), a total of 24 species were recorded (one exclusive), with 21 in the main channel and 14 in the tributaries. The most abundant species captured using the passive method was *H. albopunctatus* (44.4%). In contrast, the active method revealed that *Bryconamericus ikaa* was the most abundant species in the main channel (26.4%), whereas *P. troya* was the most abundant in the tributaries (33.1%). The upper region of the basin (sampling sites C5, C6, T5 and T6) exhibited lower richness and diversity, with only 21 species recorded in total (one exclusive), 19 in the main channel and 10 in the tributaries. In this region, the most abundant species captured by the passive method was ‘*G*’. *iporangensis* (33.3%). When using the active method, *A. lacustris* was the most abundant species in the main channel (36.8%), whereas *P. troya* dominated the tributaries (76.5%). Considering the three regions, 20 species occur in all of them.

**FIGURE 10:**
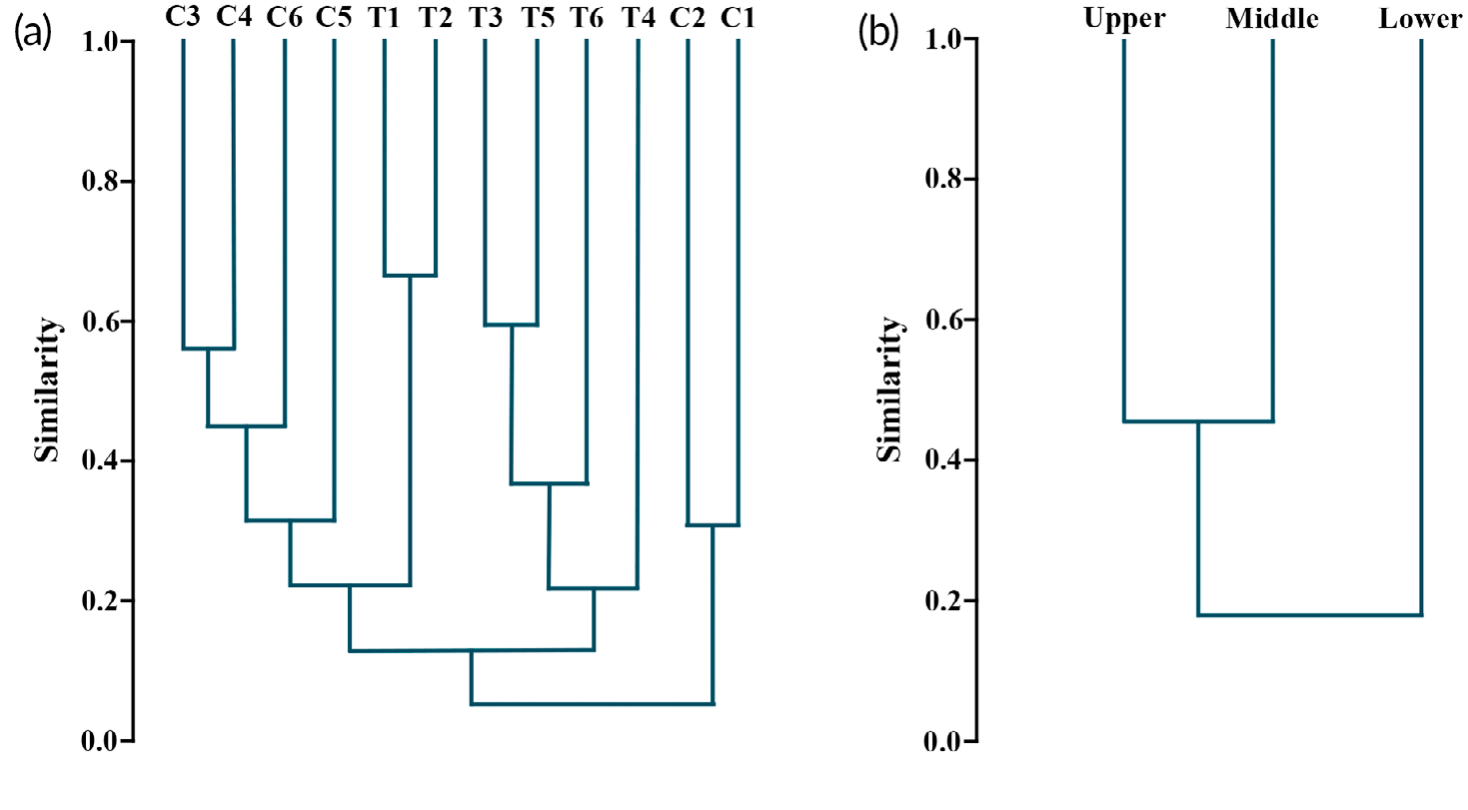
Similarity (Bray–Curtis index) between sample sites (a) and altimetric regions (b) in the São Francisco Braço Norte basin.

Species composition at sampling sites in the main channel of the lower region (C1 and C2) was significantly different from the other sites (Figure 10a; Table 2). Even among these sites, similarity was low (0.31), primarily due to the many species unique to site C1. The other sampling sites in the main channel were grouped, with the most significant similarity observed between sites C3 and C4. Regarding the tributaries, species composition at the sampling sites in the lower region (T1 and T2) was similar but significantly different from the other sites (Figure 10a). Similarly, sampling sites T3 and T5 were more similar to each other and weakly grouped with sampling sites T6 and T4. Considering the altimetric regions in the basin, the three regions presented low similarity (<0.5), with the lower region being markedly distinct from the middle and upper regions (0.18).

**FIGURE 11:**
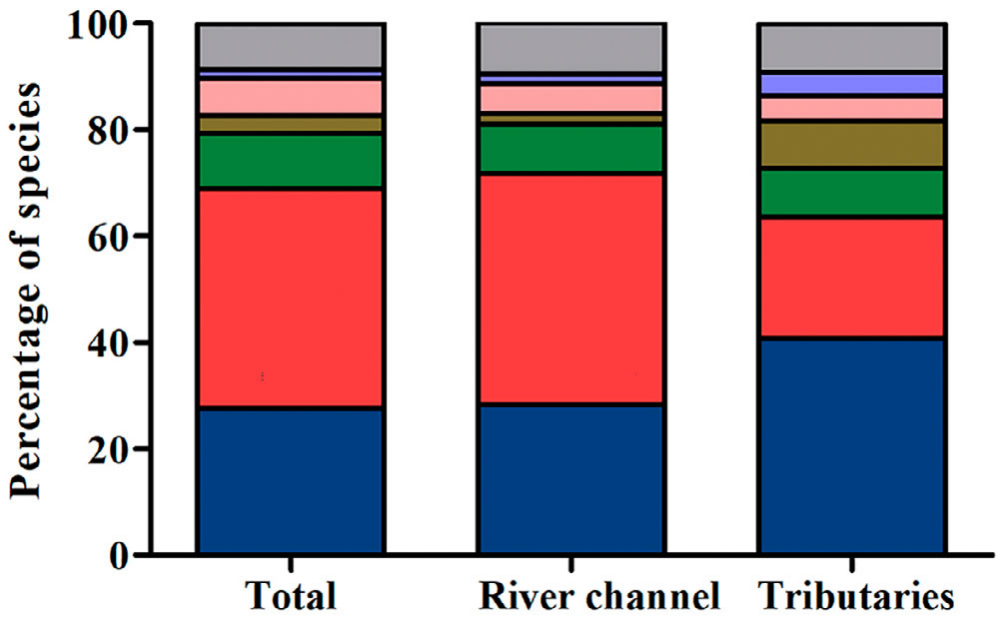
Percentage of fish species collected in the São Francisco Falso Braço Norte River basin based on their geographic origin. Blue: natural distribution in the Paraná River basin both above and below the Sete Quedas Falls. Red: natural distribution in the Paraná River basin just below the Sete Quedas Falls. Green: natural distribution in the Paraná River basin only above the Sete Quedas Falls. Brown: previously considered endemic to the Iguassu River basin; species native to other Neotropical regions and non‐native in the Paraná River basin (light pink); species native to another continent (purple); species with uncertain origins in the study area (grey).

According to the IUCN for extinction risk, *P. troya* is classified as vulnerable (VU), whereas *Crenicichla mandelburgeri* is categorized as near threatened (NT). Most other species are listed as least concern (LC) (39; 67.2%). Additionally, 17 species (29.3%) have not been assessed, primarily due to taxonomic uncertainties. In terms of species origin, 16 species (27.6%) are originally found in the Paraná River basin both above and below the Sete Quedas Falls; 24 (41.4%) have a natural distribution in the Paraná River basin just below the Sete Quedas Falls; six (10.3%) have an original distribution in the Paraná River basin only above the Sete Quedas Falls; two species (3.6%) were previously considered endemic to the Iguassu River basin; four (6.9%) are native to other Neotropical regions but are non‐native to the Paraná River basin; one species (1.7%) is native to another continent; and five (8.6%) have uncertain origins within study area (Table 2; Figure 11).

## 4 DISCUSSION

This study recorded 58 species in the SFFBN River basin. It represents 90.6% of the estimated richness (64 species), reliably covering the diversity of this basin. The highest richness was observed in the orders Characiformes (48.3%) and Siluriformes (27.6%), reflecting a general pattern of regional diversity among Neotropical freshwater fish (Lowe‐McConnel, 1999) and specifically within the area influenced by the Itaipu Reservoir (Agostinho et al., 1994; Benedito‐Cecilio & Agostinho, 2000). According to the more recent investigation of South American fish diversity conducted by Reis et al. (2016), the orders Characiformes and Siluriformes account for approximately 33% and 37% of all species, respectively. Furthermore, the high representativeness of Acestrorhamphidae, Characidae and Stevardiidae (21 species in total; 36.2%) identified in this study underscores the significance of these small characins in the study area. Their diversity and abundance may be related to the smaller size of the main channel and the inclusion of low‐order tributaries in the sampling, as the significant representation of these taxa is a general evolutionary pattern for the ichthyofauna of South American streams (Castro, 2021).

Studies of the ichthyofauna in the Paraná River, particularly in the region of the Itaipu Reservoir, began in 1977 (Itaipu Binacional, 1981). Before the damming of the Paraná River by the Itaipu Dam in 1982, 113 fish species were recorded in the stretch ranging from the area immediately downstream of the Sete Quedas Falls to the Itaipu Hydroelectric Dam, of which approximately 83 were identified in the reservoir about 4 years later – *Hypostomus* spp. counted as a single unit before and after the impoundment (Agostinho et al., 1994). In addition to the loss of certain species in the dammed portion, the primary changes in the ichthyofauna resulting from the damming involved alterations in the community structure, including shifts in the most abundant species and predominant trophic categories (see Agostinho et al., 1992, 1994; Agostinho & Júlio Júnior, 1999; Benedito‐Cecilio & Agostinho, 2000; Benedito‐Cecílio et al., 1997). Notably, there was a significant increase in species diversity in the main tributaries on the left bank of the reservoir. Before the Itaipu Reservoir was filled, sampling in the lower stretch of the São Francisco Falso River yielded only nine fish species, with *Hypostomus* spp. counted as a single unit (Benedito‐Cecílio et al., 1997). However, after the damming, species richness increased to approximately 30 species, indicating that species previously confined to the Paraná River dispersed into this tributary (Benedito‐Cecílio et al., 1997). Compared to the previous species list compiled specifically for the São Francisco Falso River basin following damming (Benedito‐Cecílio et al., 1997), this study reveals a remarkable 93.3% increase in the number of recorded species solely in the Braço Norte region.

The Itaipu Reservoir and the lower stretch of its main tributaries, including the Rio São Francisco Falso, have been extensively studied since the formation of the reservoir (e.g., Agostinho et al., 1994; Agostinho & Júlio Júnior, 1999; Benedito‐Cecilio & Agostinho, 2000; Benedito‐Cecílio et al., 1997). The findings regarding the taxonomic composition of the ichthyofauna in the Itaipu Reservoir and the floodplain of the Upper Paraná River, located upstream, were compiled by Graça and Pavanelli (2007) and later updated by Ota et al. (2018), encompassing a total of 211 fish species. Reis et al. (2020) gathered information on fish diversity in the state of Paraná, Brazil, documenting 154 species of fish in the Paraná River and its tributaries below the Sete Quedas Falls (excluding the Iguassu River upstream of the Falls). Pereira et al. (2021) followed the identification key published by Ota et al. (2018) and used molecular data to identify the ichthyofauna of streams within the watersheds on the Brazilian side of the Itaipu Reservoir. These researchers sampled 48 fish species and reported cryptic species identified through molecular analysis. More recently, Dagosta et al. (2024) published a comprehensive review of fish diversity across the entire Upper Paraná ecoregion, documenting 341 native species (above the Sete Quedas Falls) and 128 non‐native species, of which 49 are attributed to the Itaipu Reservoir and 10 to the Piracema Channel. In other words, almost half (46%) of non‐native species of the Upper Paraná River basin are a consequence of the Itaipu's building.

Despite these recent reviews, this study on the SFFBN River basin presents five new records of species for the Upper Paraná ecoregion (sensu Abell et al., 2008), specifically *Crenicichla* cf. *gillmorlisi*, *C. mandelburgeri*, *P. troya, B. ikaa* and *Hoplisoma* cf. *carlae*. Besides, *C*. cf. *gillmorlisi* and *P. troya* also represent new records for Brazil. All of them occur only in sampling points in stretches of the SFFBN River which are not under direct influence of the Itaipu Reservoir. It indicates they may be native, and apparently, the lentic environment of the Itaipu Reservoir prevented them from dispersing to other regions of the Upper Paraná ecoregion. The first three are known from the Middle Paraná River basin, whereas the last two were previously considered endemic to the Iguassu River basin.

Before the flooding, the Sete Quedas Falls consisted of a series of large cascades through which water flowed in a canyon measuring 5 km in length, 60 m in width and 100 m in height, acting as an ecological filter and separating two distinct ichthyofaunal communities (Bonetto, 1986; Vitule et al., 2012). According to Oliveira et al. (2004, 2005), the damming of the Paraná River to fill the Itaipu Reservoir has led to increased homogenization of the ichthyofauna along the reservoir's shores. However, higher values of β‐diversity in the lentic and lotic stretches of the tributaries indicate that the ichthyofaunal communities of these rivers remain partially distinct from those of the reservoir and from one another (Oliveira et al., 2004, 2005). The new records mentioned above suggest that, despite being currently integrated with the Upper Paraná ecoregion and significantly influenced by the dynamics of the Itaipu Reservoir, the fish community of the SFFBN River basin still harbours species that may be linked to the region's biogeographic history. This is supported by the fact that we found only six species (10.6%) naturally distributed in the Paraná River basin exclusively above the Sete Quedas Falls, which occur within the SFFBN: *Schizodon nasutus, Psalidodon bockmanni, Hoplias* aff. *malabaricus* (referred to as *Hoplias* sp. 2 in Ota et al., 2018), ‘*G*.’ *iporangensis, Saxatilia britskii* and *Hypostomus strigaticeps*. All of them are highly environmentally tolerant and were successful in colonizing the SFFBN River basin after the Sete Quedas Falls were submerged.

On the contrary, some species considered endemic above Sete Quedas Falls were putatively believed to be native to the SFFBN River basin here due to records documented below Sete Quedas Falls before the formation of the reservoir. These records, extracted from the literature, include *Oligosarcus paranensis* (see the list of type material in Menezes & Gery, 1983) and *Serrapinnus notomelas*, which was captured in the Puerto Bertoni region of the Alto Paraná Department, Paraguay, in 1982 (Uj, 1987). However, these records were not confirmed in this study and warrant further investigation in the future. *Pterygoplichthys ambrosettii* is recognized as native of the Paraná River basin below Sete Quedas. Nevertheless, the absence of this species in early surveys suggests that there was likely no resident population in the area that today corresponds to the Itaipu Reservoir (Silva et al., 2022). According to these authors, the aquarium hobby is likely responsible for the introduction of this species in the Itaipu Reservoir and the Upper Paraná River basin.

Another noteworthy record is that of *Deuterodon luetkenii*, whose presence in the study area is linked to historical and current connectivity between the Middle Paraná River basin and Upper Paraná ecoregion. This species was previously reported in the Itaipu Reservoir region by Reis et al. (2020) but is absent in the studies conducted by Ota et al. (2018), Pereira et al. (2021) and Dagosta et al. (2024). The first known record of *D. luetkenii* in the stretch between the Itaipu Dam and the Sete Quedas Falls was in a stream in the Municipality of Santa Helena, state of Paraná, in 1989 (NUP 2621). This suggests that a population of this species already inhabited this stretch prior to the construction of the Piracema Channel, providing evidence that *D. luetkenii* may be native to the area under study. The geographic distribution of *D. luetkenii* reported in the literature includes the basins of Paraguay, Uruguay and Paraná rivers (below Sete Quedas Falls) (Almirón et al., 2015; Carvalho et al., 2022; Loureiro et al., 2023), as well as coastal rivers of the Atlantic Forest in Rio de Janeiro, Paraná and Rio Grande do Sul (Fricke et al., 2023; Reis et al., 2020). In this study, *D. luetkenii* was captured exclusively at point C1 in the SFFBN River basin. However, an analysis of material deposited at the CISH revealed that *D. luetkenii* also occurs in other tributaries of the Itaipu Reservoir, such as the Rio Pacurí (CISH 140) and São Francisco Falso Braço Sul (CISH 141, 142, 143, 144, 733).

The cichlids *Geophagus sveni*, *Cichla kelberi* and *S. setepele* are native to the Araguaia‐Tocantins River basin (Kullander & Ferreira, 2006; Lucinda et al., 2010; Ota et al., 2021). These species are highly adapted to lacustrine environments and have thrived in several dammed sections of the Upper Paraná River basin (Dagosta et al., 2024). In the SFFBN River basin, these three species were captured only at sampling site C1 and in low abundance. The physiographic and limnological characteristics of the upper portions of SFFBN River may have restricted the dispersal of these species towards the lotic stretches. Therefore, preserving free‐flowing tributaries can prevent the establishment of these invasive species and conserve native species, which are strongly adapted to riverine systems (Sanches et al., 2016). In addition, the non‐native species *Oreochromis niloticus* and *Poecilia reticulata* were recorded. The presence of these species is linked to fish farming and the control of mosquito larvae, respectively (Dagosta et al., 2024; Ota et al., 2018). Non‐native species negatively impact the environment through several mechanisms and often contribute to biodiversity loss (Franco et al., 2024; Pelicice, Agostinho, Alves, et al., 2023). Furthermore, five species have uncertain origins in the study area, primarily due to taxonomic ambiguities. The only exception is *Megalamphodus eques*, whose popularity in the aquarium trade and absence in pioneering surveys of the Upper Paraná ecoregion have raised questions regarding the species' origin in that region (Dagosta et al., 2024).

The establishment of the Itaipu Reservoir flooded the lower stretches of the SFFBN River basin up to approximately 220 m (Fernandez & Baller, 2018), resulting in the expansion of lentic environments and facilitating the proliferation of lacustrine species (Agostinho et al., 1992). In the studied area, all sampling sites retain their riverine characteristics, except site C1, located near the mouth of the SFFBN River within the Itaipu Reservoir. At this site, the flow of the tributary waters is significantly slowed, leading to increased sediment deposition. The highest species richness and diversity were recorded at this site, featuring species typical of lentic environments, such as *Catathyridium jenynsii*, *C. kelberi*, *G. sveni*, *L. platymetopon*, *Potamotrygon amandae*, *S. setepele* and small characins predominantly found along the reservoir shores, including *Aphyocharax anisitsi*, *Ctenobrycon kennedyi, M. eques*, *Moenkhausia bonita*, *Bario forestii* and *Roeboides descalvadensis*. None of the above species were identified upstream in the SFFBN River basin. The C1 site represents an intermediate environment where both lentic and lotic species can coexist, utilizing the habitat temporally and enhancing local diversity (Oliveira et al., 2003, 2004).

The Bray–Curtis similarity analysis revealed an evident influence of the Itaipu Reservoir on the fish community structure in the lower stretch of the SFFBN basin. Specifically, it distinguished sampling sites C1 and C2, as well as tributaries T1 and T2, from the remaining sites (Figure 10a), highlighting the uniqueness of the ichthyofauna in this reservoir‐influenced stretch. Additionally, the lower region of the basin was notably distinct from the middle and upper stretches in terms of species abundance and composition (Figure 10b). This pattern reinforces the ecological singularity of the reservoir margins and supports the need to preserve the integrity of the middle and upper stretches of the basin, as they retain a fish fauna that is potentially more representative of the region's original biogeographic composition.

The middle and upper regions of the SFFBN River basin also had low similarity to the Bray–Curtis index (Figure 10b). To better understand whether these differences were driven by species identity or abundance, we complemented the analysis using the Jaccard index, which considers only species presence or absence (Jaccard, 1901). This exploratory analysis indicated a relatively high taxonomic similarity between the middle and upper regions (Jaccard = 0.73), suggesting that the differences detected by Bray–Curtis were primarily related to abundance patterns rather than species turnover.

The rich diversity of South American freshwater fishes is increasingly threatened by habitat loss and degradation, river fragmentation from dam construction, pollution and the introduction of non‐native species (Pelicice et al., 2021; Reis et al., 2016). Global assessments indicate an alarming 81% decline in freshwater vertebrate populations (McRae et al., 2017). These losses have disrupted species composition, ecological interactions and ecosystem resilience, compromising the services these organisms provide (Chapin III et al., 2000; Dirzo et al., 2014; Pelicice, Agostinho, Azevedo‐Santos, et al., 2023).

Considering the critical importance of conserving freshwater biodiversity, a key finding of this study is the high abundance and wide distribution of *P. troya* across several sampling sites in the middle and upper stretches of the SFFBN River basin. This is particularly noteworthy given that *P. troya* is currently listed as vulnerable (VU) by the IUCN, based on criterion B1ab(iii), which considers a restricted extent of occurrence (< 20,000 km^2^) and ongoing habitat loss due to urbanization, agriculture and damming (Liotta, 2022). These threats are clearly present in the SFFBN basin, which retains less than 10% of native vegetation (Instituto Paranaense de Desenvolvimento Econômico e Social (IPARDES), 2017), is dominated by agricultural land use (~70%) and suffers from pesticide runoff and limited sewage treatment (Cunha, 2018; Pereira & Scroccaro, 2013).

The apparent contradiction between *P. troya* threatened status and its local abundance raises important questions about its population structure, habitat specificity and the adequacy of current conservation assessments. It is possible that artificial connectivity created by the Itaipu Reservoir and the Piracema Channel has facilitated the expansion or persistence of this species in previously isolated habitats. However, without detailed studies on population genetics, demographic trends and ecological requirements, it is difficult to determine whether this local abundance reflects true population stability or a transient effect.

Therefore, we emphasize the need for a comprehensive reassessment of *P. troya*, including populations from Argentina, Brazil and possibly Paraguay. In the meantime, conserving free‐flowing tributaries such as the SFFBN by protecting riparian vegetation, rehabilitating degraded areas and preventing future damming remains a crucial strategy for maintaining populations of this and other sensitive species (Marques et al., 2018).

In contrast, *C. mandelburgeri* was recorded only at one site (C2). Although classified as near threatened (NT) under criteria B1a + 2a (Frederico, 2023), this species is widely distributed throughout the Middle Paraná River basin, including the lower Iguaçu River and non‐isolated tributaries (Piálek et al., 2019; Říčan et al., 2023). As *C. mandelburgeri* represents a species complex, genetic and taxonomic studies are essential to clarify the identity and conservation status of the population found in the SFFBN. Similar attention should be given to the newly identified population of *C*. cf. *gillmorlisi*, which has yet to be assessed by the IUCN.

## 5 CONCLUSIONS

This study assessed the fish community composition of the SFFBN River basin, a Brazilian tributary of the Itaipu Reservoir, which has experienced artificial hydrological connectivity with the Upper Paraná River basin since the construction of the dam. The results document that the fish fauna in the lotic stretches of the SFFBN River basin exhibits a blend of species from both the Middle and Upper Paraná River basins, and it is partially distinct from those associated with the reservoir's shorelines, corroborating previous studies (Oliveira et al., 2003, 2004, 2005). Although the SFFBN River basin is currently integrated into the Upper Paraná ecoregion and strongly influenced by the dynamics of the Itaipu Reservoir, its fish community still houses species likely linked to the biogeographic history of the Middle Paraná River basin. These species, particularly in the middle and upper stretches of the basin, appear to have persisted without being directly affected by the damming. Additionally, five new records of species for the Upper Paraná ecoregion were documented, including two not previously recorded in Brazil, emphasizing the biogeographic and conservation significance of this tributary. Notably, the detection of *P. troya* as the most abundant species in several upstream sites challenges its current conservation classification as vulnerable (VU) and underscores the importance of reassessing its distribution, population structure and ecological requirements considering artificial connectivity. These findings reinforce the need for integrative conservation approaches that combine habitat protection, population monitoring and taxonomic clarification, particularly in free‐flowing tributaries. Long‐term monitoring of the fish community in the Itaipu tributaries, and strategic locations such as the Piracema Channel, is essential to assess the impacts of ichthyofaunal homogenization between Upper and Lower Paraná ecoregions and to inform regional biodiversity management and policy.

## AUTHOR CONTRIBUTIONS

**Lucas E. P. Kampfert:** conceptualization, data curation, formal analysis, investigation, methodology, visualization, writing – original draft. **João C. Maicrovicz:** investigation, methodology, writing – review and editing. **Daniel R. Blanco:** conceptualization, investigation, methodology, resources, supervision. **Denise Lange:** conceptualization, data curation, formal analysis, methodology, supervision, visualization, writing – review and editing. **Carla S. Pavanelli:** conceptualization, writing – review and editing. **Heleno Brandão:** conceptualization, funding acquisition, investigation, methodology, project administration, resources, supervision, writing – review and editing.

## FUNDING INFORMATION

Conselho Nacional de Desenvolvimento Científico e Tecnológico (CNPq) process # 307124/2023‐1 to Carla S. Pavanelli and # 402670/2016‐7 to Heleno Brandão.
